# Scrotal Pain in Familial Mediterranean Fever: A Series of Three Case Reports and Literature Review

**DOI:** 10.31138/mjr.180425.tar

**Published:** 2025-09-15

**Authors:** Amr Bahaa, Anati Nour, Atawneh Hamza, Gharaibiah Mohammad, Altell Kenana, Ghandi Lama, Abunejma Fawzi

**Affiliations:** 1 College of Medicine, Hebron University, Hebron, West Bank, Palestine;; 2 Division of Paediatric Rheumatologist, Department of Paediatrics, Al-Ahli Hospital, Hebron, West Bank, Palestine;; 3 Division of Paediatric Rheumatologist, Department of Paediatrics, Palestine Red Crescent Hospital, Hebron, West Bank, Palestine;; 4 College of Medicine, Istinye university, Istanbul, Turkey

**Keywords:** autoinflammatory diseases, case reports, Familial Mediterranean Fever, MEFV protein, scrotal pain, testicular diseases

## Abstract

Testicular pain is a common symptom with many differential diagnoses, but it can also be an unexpected and only presenting symptom of Familial Mediterranean Fever (FMF), which is a hereditary autoinflammatory disorder derived by cytokines, primarily characterised by recurrent episodes of fever, abdominal pain, and arthritis. In this case series, we report three male patients who initially sought medical attention for testicular pain, which was their only complaint. Some of these patients do not initially exhibit the typical presentation of FMF, making the diagnosis slightly challenging. Through comprehensive diagnostic evaluation, including genetic testing for mutations in the MEFV gene and assessment of inflammatory markers, FMF was confirmed in all the cases. These findings underscore the importance of considering FMF in the differential diagnosis of testicular pain, particularly in individuals with a family history of this disease or Mediterranean ancestry, in order to facilitate early diagnosis and appropriate treatment. Recognising this unusual presentation of FMF can help avoid misdiagnosis and improve patient outcome and prognosis.

## INTRODUCTION

Familial Mediterranean fever (FMF) is an autoinflammatory autosomal recessive disorder that is the most prevalent inherited fever syndrome. It triggers recurrent crises of fever and serosal inflammation and is caused by a mutation in the MEFV gene on chromosome 16, which encodes the pyrin protein.^1^

Approximately 300 different mutations in MEFV have been identified, which may lead to FMF.^2^ These mutations can result in symptoms such as pleurisy, occasional pericarditis, erysipeloid, myalgia, preterm delivery, and acute scrotum. The attacks usually resolve on their own within a few days, and patients often return to their usual state of health between episodes. However, if left untreated, the condition can progress to secondary AA amyloidosis, which can be fatal.^3^ Colchicine has been used for over 40 years in the treatment of FMF to reduce the frequency and severity of episodes as well as to prevent amyloidosis; however, its effectiveness may vary among individuals.^4^

Turks have the highest prevalence among populations affected by FMF, ranging from 1 in 150 to 1 in 1,000 individuals. Armenians follow with a reported prevalence of 1 in 500 patients.^5^ Additionally, FMF is prevalent in Arabic countries, with Jordan showing an estimated prevalence of 1 in 2,600 and a genetic frequency of 1 in 50.^6^

Acute scrotum, characterised by sudden onset of scrotal pain, swelling, and redness, it’s a urological emergency condition that requires prompt evaluation and intervention due to the risk of testicular torsion.^7^ However, in the case of FMF, the acute scrotum may be an inflammatory manifestation of the disease rather than an acute surgical issue. It is crucial to distinguish between FMF-related acute scrotum and other potential causes such as testicular torsion or epididymitis to prevent unnecessary surgical procedures and ensure proper management of the underlying autoinflammatory condition.^8^

Acute scrotal inflammation in children with FMF is not widely recognised. It is sometimes present alone or with an abdominal attack (peritonitis).^9^ Scrotal swelling usually presents as a painful, unilateral enlargement upon examination, which caused by temporary inflammation of the tunica vaginalis and usually resolves within 24h.^10^ Although testicular torsion may occur in patients with FMF,^10^ the first challenge in managing acute scrotal swelling to make a prompt and accurate diagnosis to prevent serious complications, with the greatest risk being a missed diagnosis of spermatic cord torsion.

This case series is significant because of the rarity of scrotal pain in FMF as a presentation and the limited research on it. Understanding its causes, symptoms, and underlying mechanisms could improve its diagnosis, treatment, and prognosis. This research will help fill this gap in the literature and expand our understanding of FMF.

## CASE PRESENTATION

### Case 1

An 11-year-old male student with a known history of FMF, diagnosed at the age of five, presents with recurrent unilateral testicular pain that began two years ago. The episodes occur every four months, with the most recent attack occurring one month ago. The pain was sharp, severe, debilitating, and confined to bed for one–two weeks per episode. It is unilateral, non-radiating, and worsens with movement, accompanied by nausea, but no fever. During these attacks, there was scrotal swelling and tenderness, although there was no skin discoloration. Despite receiving ibuprofen, intravenous fluids, and another unknown medication, the pain persisted and resolved spontaneously after two weeks, the patient did not notice significant relief with medication.

The patient had a strong family history of FMF, and an 18-year-old brother was diagnosed at 13 years of age. There was no known family history of genetic conditions or acute scrotal pain. His initial FMF symptoms included upper and lower limb pain that was unresponsive to analgesia, along with arthralgia. His FMF attacks were triggered by exercise and exposure to cold. Genetic testing revealed a homozygous M694V allele mutation in the MEFV gene. He is currently receiving Colchicine (1.5 mg daily), Naproxen (250 mg in addition to colchicine), and Prednisolone (20 mg for 3–5 days during severe attacks). The patient also received intra-articular Triamcinolone Acetonide injections for hand joint swelling. He typically responds well to Colchicine and requires additional medication only during severe episodes. There had no history of FMF-related amyloidosis.

On general examination, he appeared well, with no distress or discomfort, although pallor was noted. His vital signs were stable, and there was no fever. Abdominal examination revealed no tenderness, guarding, rebound tenderness, hepatosplenomegaly, or bowel abnormality. On scrotal examination, asymmetry due to swelling was noted, but there was no skin discoloration or visible lesions. The testis was tender with epididymal swelling. Although the cremasteric reflex was intact, Prehn’s sign and transillumination test were negative. The inguinal region was normal with no lymphadenopathy or hernia.

Musculoskeletal examination revealed joint tenderness and swelling, especially in the large joints, with possible enthesitis due to chronic muscle and bone pain. Skin examination revealed FMF-related rashes, described as warm, diffuse, erysipeloid-like rash appearing every 1–2 months, primarily on the lower extremities. It is associated with eye symptoms, including red sclera with a burning sensation.

Given the recurrent and self-limiting nature of his acute scrotal pain, along with his underlying FMF, the most likely aetiology is FMF-associated vasculitis, epididymitis, or inflammation, rather than classical testicular torsion. Further evaluation with scrotal ultrasound revealed epididymoorchitis.

Therefore, the management of orchitis presentation in patients with FMF in addition to Colchicine commitment encouragement (NSAIDS and Corticosteroids in severe attacks).

### Case 2

An 8-year-old boy presented with a history of scrotal pain bilaterally. The pain was intermittent and was associated with scrotal redness, swelling, and haematuria. He also experienced nausea and abdominal pain, but denied having fever. The pain worsened with movement and was managed with analgesics, although it was self-limiting.

The patient was diagnosed with FMF in 2023 after presenting with a purpuric rash that initially appeared on his legs before spreading to the entire body except for the face. His FMF attacks were infrequent, with only one recorded episode triggered by a streptococcal throat infection. Genetic testing revealed heterozygous E148Q and M694V allele mutations in the MEFV gene. The patient is currently receiving colchicine (1 mg daily). His family history was significant for FMF, with his 3-year-old sister and maternal cousin diagnosed with the condition.

Upon general examination, the patient appeared pale. The patient’s vital signs were stable. Abdominal examination revealed no distension, scars, hernias, or tenderness. There were no signs of hepatosplenomegaly, and bowel sounds were normal. Scrotal examination revealed bilateral swelling, redness, and enlargement, but no visible masses or skin changes. The testicles were tender bilaterally with associated epididymal swelling. Further assessments, including the cremasteric reflex, were intact, but the Prehn’s sign and transillumination test were negative.

The patient required hospitalisation during the course of illness. He had received a blood transfusion 28 days prior to admission. His first hospital admission lasted one month, and he was treated with corticosteroids for purpura, abdominal pain, and testicular swelling with haematuria, but no fever. After 15 days, the patient was readmitted for another 15 days due to recurrent symptoms. Following discharge, the patient remained symptom-free for seven days before stabilising on colchicine without further episodes.

Musculoskeletal examination results were unremarkable for joint tenderness or swelling. However, skin examination showed a purpuric rash, suggestive of vasculitis, along with erysipelas-like erythema. Inguinal examination results were normal with no lymphadenopathy or hernia.

At present, the patient is stable on Colchicine, with no recurrent symptoms.

### Case 3

A 12-year-old boy presented with a history of recurrent abdominal pain with high-grade fever for 4 years ago, it was severed in nature in the form of an episode lasted for 3–4 days, although it was self-limited. The patient also complained of 4 episodes of bilateral scrotal pain, which worsened with movement and was managed with analgesics, rash, and limping.

The patient was diagnosed with FMF in 2022 after presenting with a purpuric rash that initially appeared on his legs before spreading to the entire body except for the face. His FMF attacks have been infrequent, with only one recorded episode, which was triggered by a streptococcal throat infection. Genetic testing revealed a homozygous M694V alleles mutation on MEFV gene. He is currently on Colchicine 1 mg daily. He is the third of five healthy siblings, and there has a positive family history of fathers’ cousins.

Upon general examination, the patient complained of pain. His vital signs were stable. Abdominal examination showed no distension, scars, hernias, or tenderness. There were no signs of hepatosplenomegaly, and bowel sounds were normal. Scrotal examination revealed bilateral swelling, redness, and enlargement, but no visible masses or skin changes. The testicles were tender bilaterally. Further assessments, including the cremasteric reflex was intact, but Prehn’s sign and Transillumination test are negative. Musculoskeletal examination was unremarkable for joint tenderness or swelling. However, skin examination showed a purpuric rash on his entire body, except for the face, inguinal examination was normal, with no lymphadenopathy or hernia.

At present, the patient is stable on Colchicine with no recurrent symptoms.

## DISCUSSION

FMF is a common monogenic autoinflammatory disease that belongs to a relatively new category of disorders characterised by abnormally heightened inflammation. This inflammation is primarily driven by cells and molecules of the innate immune system and is significantly influenced by genetic predispositions.^11^ Monogenic autoinflammatory diseases, often referred to as periodic fever syndromes, are characterised by recurring, self-limiting episodes of fever and inflammation. These episodes are typically accompanied by symptoms that mimic acute abdomen. The combination of symptoms, along with the duration of episodes, forms the basis for the clinical diagnosis of this condition.^12^

FMF classically presents with recurrent fever, peritonitis, pleuritis, arthritis, and scrotal attacks, which are lesser-known manifestations that can mimic surgical emergencies such as testicular torsion or epididymo-orchitis.^13^

FMF is linked to mutations in MEFV, which encodes pyrin, a cytosolic protein with five crucial functional domains.^13^ MEFV consists of ten exons and is primarily expressed in granulocytes, monocytes, and other cells involved in FMF. Most mutations occur in exon 10, including the well-known M694V, V726A, M680I, and M694I variants. A prominent hypothesis proposes that pyrin interacts with ASC and caspase-1, preventing the activation of IL-1β and inflammatory response. Mutations in MEFV impair pyrin function, leading to un-checked IL-1β activation and inflammation.^14^

**Table 1.** Comparative clinical and genetic features of three children with FMF.

**Feature**	**Case 1**	**Case 2**	**Case 3**
**Age**	11 years old	8 years old	12 years old
**Ethnicity**	Arab	Arab	Arab
**FMF Diagnosis**	Diagnosed at 5 years old	Diagnosed at 6 years old	Diagnosed at 9 years old
**Genetic Mutation**	Homozygous M694V allele	Heterozygous E148Q and M694V allele	Homozygous M694V allele
**Family History**	Brother diagnosed at 13 years old	Sister (3 years old) and maternal cousin	Father’s cousins have FMF
**Scrotal Pain**	Recurrent, unilateral, severe, every 4 months, lasting up to 2 weeks	Bilateral, intermittent, lasted 1 month, associated with hematuria	Bilateral, recurrent (4 episodes), worsened with movement
**Associated Symptoms**	Nausea, no fever, scrotal swelling, tenderness	Nausea, abdominal pain, purpuric rash, hematuria, scrotal redness and swelling	Abdominal pain, high-grade fever, rash, limping
**Response to Colchicine**	Mild respond, and requires additional medications during severe episodes	Stabilized on Colchicine after hospitalization	Stable on Colchicine, no recurrent symptoms
**Scrotal Exam**	Asymmetry, tenderness, epididymal swelling	Bilateral swelling, redness, tenderness	Bilateral swelling, redness, tenderness
**Hospitalisation**	No	Yes (1-month total, 2 admissions)	No
**Other Findings**	FMF-related rashes, joint pain, eye symptoms	Purpuric rash, erysipelas-like erythema, HSP	No
**Exclusion of Behcet disease**	Excluded	Excluded	Excluded

Acute scrotal pain in paediatric and adolescent males is a significant clinical presentation that requires urgent evaluation to exclude testicular torsion, epididymitis, or other serious urological conditions. However, when a patient presents with recurrent scrotal pain and has a personal or family history of FMF, clinicians should consider FMF as a possible underlying cause. The cases presented in this report highlight FMF as an often overlooked yet important differential diagnosis in male patients experiencing recurrent scrotal pain.

The Eurofever Criteria, a widely accepted diagnostic framework for FMF, were applied in these cases. Patients with a typical phenotype and genetically confirmed mutations of FMF are defined as phenotype I, whereas phenotype II patients develop amyloidosis without any previous attacks typical of FMF.^8^

Although acute scrotum has been recognised as a feature of FMF in Jewish and Arab patients, it can also be a clinical feature in Turkish patients with FMF. Early diagnosis and colchicine prophylaxis are essential to prevent amyloidosis, the most significant, unique, and lethal complication of FMF. Around the age of 40 years, amyloidosis is seen in 90% of untreated FMF patients. These patients undergo unnecessary interventions and recurrences, which lead to a risk of ischemic testicular necrosis.^8^

In these cases, the homozygous M694V mutation was identified, which is known to be associated with severe FMF phenotypes, earlier disease onset, and higher rates of complications such as amyloidosis. The first patient, an 11-year-old male diagnosed with FMF at the age of 5 years, experienced recurrent unilateral scrotal pain every four months, lasting 1–2 weeks, with associated tenderness and swelling but without redness or fever. His symptoms worsened with movement, were poorly responsive to analgesics, and eventually resolved spontaneously. The second patient, an 8-year-old male recently diagnosed with FMF, had one episode of bilateral scrotal pain lasting one month, accompanied by swelling, redness, and haematuria. His episode was associated with Henoch-Schönlein Purpura (HSP), a vasculitic condition known to overlap with FMF. The third patient, a 12-year-old girl diagnosed with FMF, had recurrent episodes of abdominal pain, high grad fever, and bilateral scrotal pain lasting 3–4 days, accompanied by swelling, redness, and a general purpuric rash except his face.

FMF is characterised by recurrent episodes of inflammation in various parts of the body, including the testicles in males. This inflammation leads to scrotal pain, which is a manifestation of periodic attacks that also affect the abdomen, chest, and joints. This condition is caused by genetic mutations that result in the abnormal regulation of the immune system, leading to inflammatory episodes. These episodes can be triggered by various factors, and the pain typically resolves within 12–72 h.^15^

Recognising FMF as a cause of recurrent scrotal pain is vital to avoid unnecessary surgeries, such as orchidopexy or orchiectomy. Clinical features suggestive of FMF over torsion or infection include recurrent, self-resolving scrotal pain, absence of signs of systemic infection, positive family history or consanguinity, associated FMF symptoms (e.g., periodic fever, arthritis, peritonitis), and a positive response to colchicine therapy. In the cases discussed, colchicine was essential for long-term management, either alone or with NSAIDs or corticosteroids during severe episodes. Early initiation of colchicine helps reduce attack frequency, stabilise symptoms, and prevent serious complications such as amyloidosis.

## CONCLUSION

These three cases emphasise the importance of recognising FMF as a potential cause of acute and recurrent scrotal pain in male patients with a relevant family history. Clinicians should maintain a high index of suspicion for FMF in endemic populations and avoid unnecessary surgical intervention. Early diagnosis and initiation of colchicine therapy are essential to prevent recurrent inflammatory episodes and improve long-term outcomes. Further research is required to better understand the pathophysiology of FMF-associated scrotal pain and swelling.

## AUTHOR CONTRIBUTIONS

Amr Bahaa: Conceptualisation, Methodology, Writing – Original Draft, Supervision.

Anati Nour: Formal Analysis, Visualisation, Writing – Review & Editing.

Atawneh Hamza: Project Administration, Data Curation, Writing – Review & Editing.

Gharaibiah Mohammad: Data Collection, Validation, Writing – Review & Editing.

Altell Kenana: Literature Review, Resources, Writing – Review & Editing.

Ghandi Lama: Investigation, Methodology, Writing – Review & Editing.

Abunejma Fawzi: Supervision Methodology, Writing – Review & Editing.

All authors take full responsibility for the integrity and accuracy of all aspects of the work.

## FUNDING

The author(s) received no financial support for the research, authorship, and/or publication of this article.

## CONFLICT OF INTEREST

The author(s) declared no potential conflicts of interest with respect to the research, authorship, and/or publication of this article.

## INFORMED CONSENT

Written informed consent was obtained from all the patient(s) parents/legal guardians for the anonymised information to be published in this article.

## ETHICAL APPROVAL

Our constitution doesn’t require IRB for case series studies.

## RESEARCH REGISTRATION UNIQUE IDENTIFYING NUMBER (UIN)

Not applicable.
